# Disorganization of chondrocyte columns in the growth plate does not aggravate experimental osteoarthritis in mice

**DOI:** 10.1038/s41598-020-67518-0

**Published:** 2020-07-01

**Authors:** Ana Lamuedra, Paula Gratal, Lucía Calatrava, Víctor Luis Ruiz-Perez, Raquel Largo, Gabriel Herrero-Beaumont

**Affiliations:** 1 Bone and Joint Research Unit, Rheumatology Department, IIS-Fundación Jiménez Díaz UAM, Reyes Católicos, 2, 28040 Madrid, Spain; 2 Instituto de Investigaciones Biomédicas ‘Alberto Sols’, CSIC-UAM, 28029 Madrid, Spain; 3 CIBER de Enfermedades Raras (CIBERER), ISCIII, Madrid, Spain; 4 Instituto de Genética Médica Y Molecular (INGEMM), Hospital Universitario La Paz-IdiPaz-UAM, 28046 Madrid, Spain

**Keywords:** Osteoarthritis, Animal disease models, Genetic models, Cartilage

## Abstract

Osteoarthritis (OA) is a multifactorial joint disease mainly affecting articular cartilage (AC) with a relevant biomechanical component. During endochondral ossification growth plate (GP) chondrocytes arrange in columns. GPs do not ossify in skeletally mature rodents. In neonatal mice, an altered joint loading induces GP chondrocyte disorganization. We aimed to study whether experimental OA involves GP disorganization in adult mice and to assess if it may have additional detrimental effects on AC damage. Knee OA was induced by destabilization of the medial meniscus (DMM) in wild-type (WT) adult mice, and in Tamoxifen-inducible Ellis-van-Creveld syndrome protein (*Evc)* knockouts (*Evc*^*cKO*^), used as a model of GP disorganization due to Hedgehog signalling disruption. Chondrocyte column arrangement was assessed in the tibial GP and expressed as Column Index (CI). Both DMM-operated WT mice and non-operated-*Evc*^*cKO*^ showed a decreased CI, indicating GP chondrocyte column disarrangement, although in the latter, it was not associated to AC damage. The most severe GP chondrocyte disorganization occurred in DMM-*Evc*^*cKO*^ mice, in comparison to the other groups. However, this altered GP structure in DMM-*Evc*^*cKO*^ mice did not exacerbate AC damage. Further studies are needed to confirm the lack of interference of GP alterations on the analysis of AC employing OA mice.

## Introduction

Osteoarthritis (OA) is a joint disease with multifactorial etiology, affecting all joint tissues, specially articular cartilage (AC), which progressively undergoes erosion and loss of its mechanical properties^1–3^. It is a chronic disease associated with aging^4^ that displays a low-grade inflammation in the affected joints, causing chronic pain and physical and functional disability^2,5^. In fact, it is the most frequent and disabling rheumatic pathology, with a prevalence of 33% in the population over 65 years of age^2,6^. Current treatments relieve some symptoms of the disease, such as pain, although they do not alter the natural course of the disease^5^. Pathogenic mechanisms of tissue damage of a purely mechanical origin coexist with biological processes of inflammatory and proliferative nature^7^. The etiopathogenic factors responsible for the initiation and progression of OA can be classified into three main axes: biomechanical alterations, local low-grade chronic inflammation and the obesity/metabolic syndrome axis^5^. However, the pathophysiology of OA is still unclear, and preclinical and clinical research is very active in order to improve our understanding of the onset and progression of the disease.

A great variety of animal models, from rodents to larger animals, are used, trying to mimic the human disease and study its pathogenesis. The mouse model of OA induced by surgical destabilization of the medial meniscus (DMM) suitably reflects human OA features, and it is the most commonly selected approach^8–13^. The skeletal growth of the mouse concludes despite the growth plates (GP) of long bones do not ossify, which impedes the fusion of the epiphysis with the metaphysis^9,14–18^. This particular characteristic of rodents constitutes a substantial difference between these joints and the human ones. However, it has not been described whether this apparently minor anatomical feature could in some way call into question some observations in rodent models of OA. In fact, around 80% of the studies using mouse models are not reproducible in humans^19^. Although the GP disappears in the human adult joint, its particular presence in mice is a relevant fact that demands further research due to the extensive use of this experimental model in the study of the pathophysiology of OA.

Limb bone formation occurs through endochondral ossification. In the GP, chondrocytes sequentially align as they differentiate. This process is mediated by several synchronized signalling pathways, including Wnt and Notch signalling, bone morphogenetic proteins, fibroblast growth factors, insulin-like growth factor 1, and the Hedgehog (Hh)–parathyroid hormone-related protein (PTHrP) axis. Hh pathway is especially relevant in orchestrating chondrocyte hypertrophy during endochondral ossification. Indian Hedgehog (Ihh) and PTHrP, coordinated in time and space, stimulate the proliferation of chondrocytes and delay their differentiation, thus preventing premature hypertrophy and ensuring a certain bone growth^20–22^. In addition, Ihh-mediated signalling activates the Glioma-Associated Oncogene (GLI) transcription factors, which induce the expression of Runx2 and promote chondrocyte hypertrophy. Therefore, there is a balance between proliferation and hypertrophy in the GP chondrocyte population dependent on negative feedback between Ihh and PTHrP action^21,22^.

The primary cilium is a mechanosensing non-motile structure that projects from the cell and plays a crucial role in the transduction of a variety of extracellular signals. After chondrocyte division in the proliferative zone, the cilium of the resulting cells orients and cells polarize^23^. Then, by successive rotational movements, chondrocytes in the GP align parallel to the longitudinal axis of the bone and stack in columns^23^. Mutations affecting ciliogenesis or the function of proteins anchored to its structure, result in a group of conditions known as ciliopathies, many of which include skeletal phenotypes^24^. Ellis van Creveld (EvC) syndrome is a ciliopathy characterized by disproportionate short stature, short limbs, narrow chest and post-axial polydactyly, among other features^24,25^. Loss-of-function mutations in *EVC* and *EVC2* cause the EvC syndrome. *EVC* and *EVC2* protein products localize at the base of the primary cilium and regulate Hh signal transduction^25^.

It has been previously demonstrated that disruption of Hh signalling by *Evc* deletion alters bone development in mice. *Evc*^*–/–*^ embryos exhibit shorter proliferative and hypertrophic chondrocyte regions in the GP, and overall, shorter bones. Also, *Evc*^*–/–*^ mice at postnatal day 16 (P16) show abnormal tibia shape and disorganized epiphyseal chondrocytes^26^. An altered biomechanical force on chondrocytes during bone development also evokes inaccurate GP function and defective bone growth^26–28^. In neonatal mice, a reduced mechanical joint loading due to hemiplegia induces the disorganization of chondrocyte columns in the GP and diminished tibial length^27^. Similarly, joint immobilization in 6-week-old rats that mimicked an unloading state, reduced the length of the tibial GP, resulting in abnormal bone growth^28^. In a different approach, in a mouse model of spondyloepiphyseal dysplasia congenita, which resulted in an increased mechanical loading on the shoulder, a loss of GP chondrocyte column organization in the humerus was described^29^.

As chondrocytes in hyaline cartilage have mechanosensors, variations in the pressure of their environment can affect the interactions between cells and with the extracellular matrix (ECM).

We aimed to study whether the induction of experimental OA could disturb GP organization in adult mice. Moreover, we wanted to assess if GP alteration triggers a further deleterious effect on AC damage. For the latter purpose, we induced the knee destabilization model of OA to tamoxifen (TAM)-induced *Evc* knockout mice, known to have altered GP chondrocyte organization.

## Results

### GP structure is altered during OA

**Figure 1:**
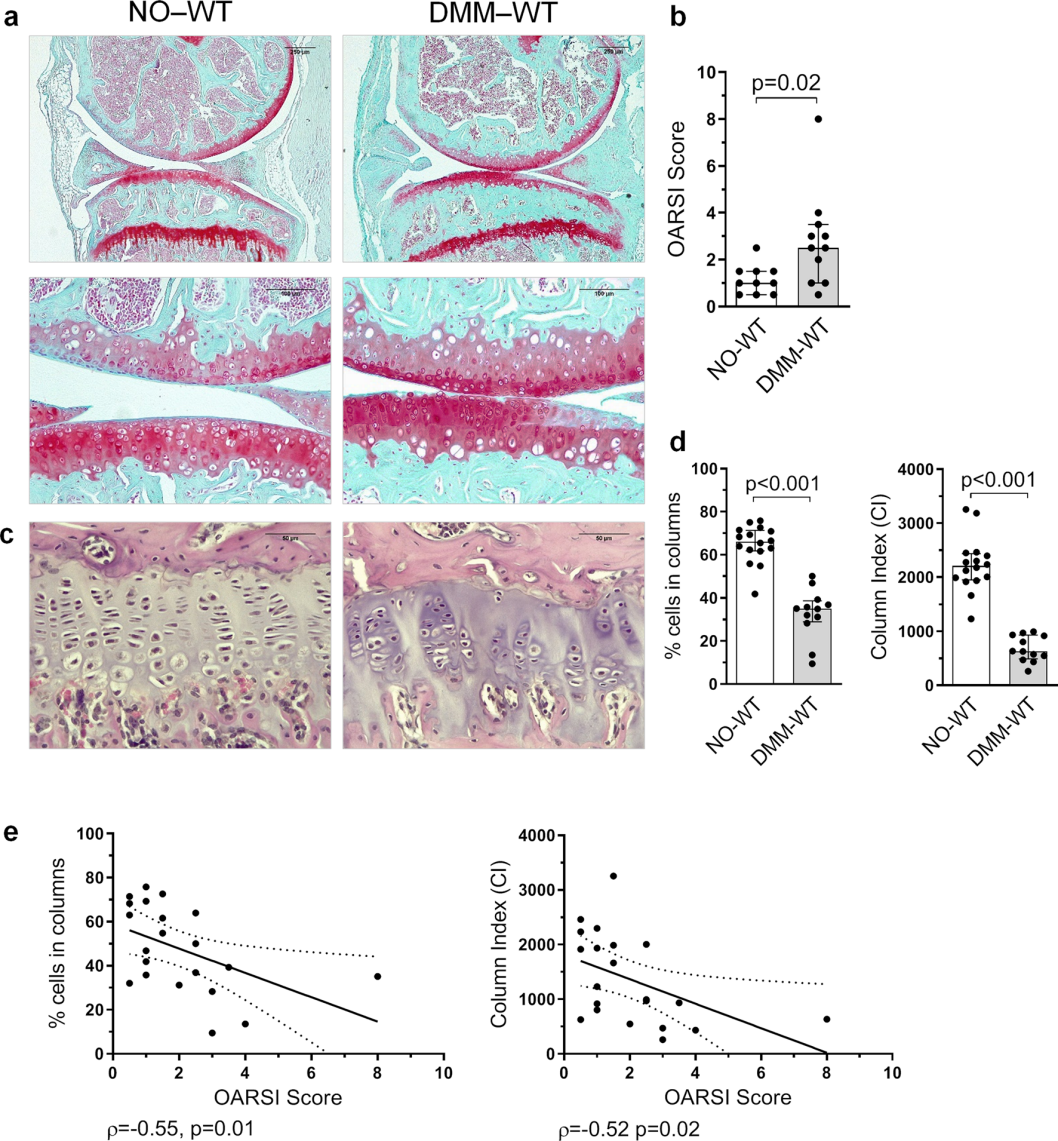
Disorganization of GP chondrocytes during experimental knee osteoarthritis (OA). Destabilization of medial meniscus (DMM) was induced in 12 weeks old mice, that were sacrificed 8 weeks after surgery. Post-traumatic damage accounted for the articular cartilage (AC) lesions, regarding proteoglycan loss, cartilage fibrillation and vertical clefts observed in the AC. (**a**) Representative mice joint sections stained with Safranin-O Fast Green at × 5 and × 20 magnification from non-operated wild type (NO-WT) and DMM-WT mice (bars = 250 and 100 µm respectively). (**b**) Histopathological OARSI Score represented as the sum of tibia and femur scores. (**c**) Representative Haematoxilin-Eosin stained tibia growth plate (GP) sections at × 40 magnification from NO-WT and DMM-WT mice (bars = 50 µm). (**d**) Percentage of cells in columns and column index (CI) in the GP in NO-WT and DMM-WT mice. Data are shown as median ± interquartile range (IQR). (**e**) Association between the percentage of cells in columns and OARSI score, and between CI and OARSI score in WT mice. NO-WT, n = 10–16; DMM-WT, n = 11–12.

AC damage was evaluated in all joints obtained at sacrifice, 8 weeks after DMM surgery. Post-traumatic damage due to surgical DMM accounted for the AC lesions observed in the DMM-WT mice, that were characterized by proteoglycan loss, cartilage fibrillation and vertical clefts. Cartilage erosions and clefts were mainly located in the bearing region of the tibia (Fig. 1a). As expected, DMM-WT mice showed a higher OARSI score than that observed in NO-WT animals (Fig. 1b). We then studied whether experimental knee destabilization was able to modify the columnar structure of chondrocytes in the GP. While NO-WT mice showed GP chondrocytes arranged in the characteristic columns, DMM-WT mice presented an altered columnar structure, and clusters of cells were observed (Fig. 1c). The percentage of cells in columns in DMM-WT animals was significantly lower than that found in the NO-WT group, as well as its CI (Fig. 1d). We observed a negative association between the percentage of cells in columns and the OARSI score (ρ = -0.55, p = 0.01), as well as between the CI and the OARSI score (ρ = -0.52, p = 0.02) (Fig. 1e). In addition, the number of columns, the mean column length, the tissue cellularity, and the number of cells per column also decreased in the DMM-WT group in comparison to NO-WT animals (Supplementary Fig. S2).

### GP chondrocyte disorganization driven by *Evc* deletion does not induce AC damage

In order to evaluate whether GP alteration per se could induce OA-like cartilage damage, we employed *Evc*^*cKO*^ mice. It has been previously observed that newborn *Evc*^*-/-*^ mice display a clear disarrangement in the GP^26^ due to Hh signalling disruption. Therefore, we used adult *Evc*^*cKO*^ mice, in which both Evc and Evc2 expression were deleted after skeletal maturity, as a model of GP disorganization in adult mice. In order to assure that potential AC lesions were not caused by bone alterations due to genetic manipulation in *Evc*^*cKO*^ mice, we first assessed bone growth and quality in these mice. *Evc*^*cKO*^ mice showed no apparent morphological defect and normal skeletal development. No differences were observed in the body weight between WT and *Evc*^*cKO*^ mice at 12 weeks of age, just before DMM surgery (Supplementary Fig. S3).

**Figure 2:**
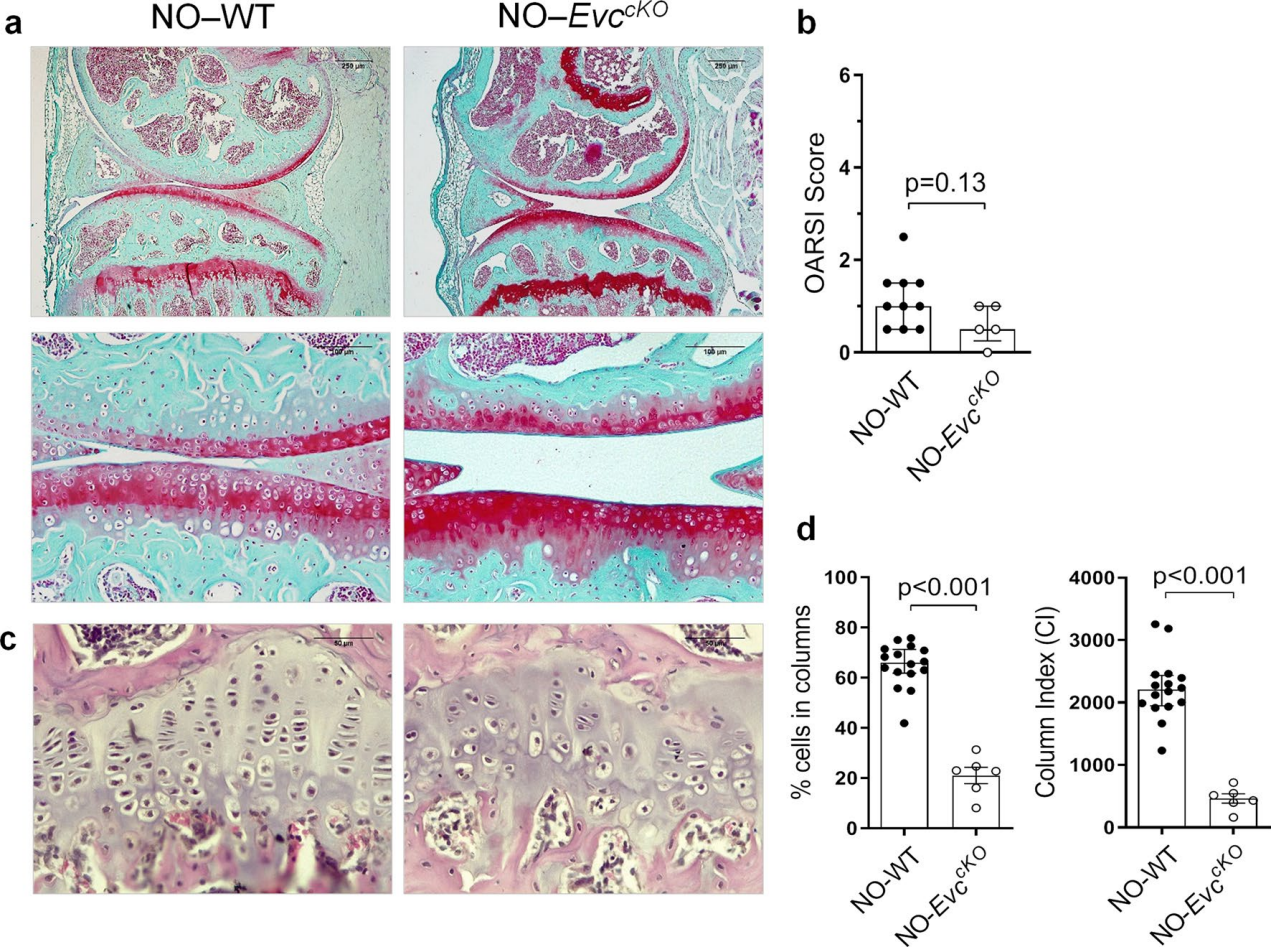
Effect of GP chondrocyte disarrangement on articular cartilage (AC) in the absence of experimental osteoarthritis (OA). (**a**) Representative Safranin-O Fast Green stained mice joint sections at × 5 and × 20 magnification in NO-WT and NO-*Evc*^*cKO*^ mice (bars = 250 and 100 µm respectively). (**b**) Histopathological OARSI Score represented as the sum of tibia and femur scores in NO-WT and NO-*Evc*^*cKO*^ mice. (**c**) Representative Haematoxilin-Eosin stained tibia growth plate (GP) sections at × 40 magnification in NO-WT and NO-*Evc*^*cKO*^ mice (bars = 50 µm). (**d**) Percentage of cells in columns and column index (CI) in the GP of NO-WT and NO-*Evc*^*cKO*^ mice. Data are shown as median ± interquartile range (IQR). NO-WT, n = 10–16; NO-*Evc*^*cKO*^, n = 5.

Consequently, we analyzed GP chondrocyte arrangement in NO-*Evc*^*cKO*^ animals, and observed that this group showed a clear GP chondrocyte disorganization in the adult stage (Fig. 2c), with a decreased CI and percentage of cells in columns in comparison to NO-WT, (Fig. 2d), as well as a lower GP cellularity, mean column length and number of cells per column than those measured in NO-WT mice (Supplementary Fig. S2). Nevertheless, NO-*Evc*^*cKO*^ animals did not show AC damage, and presented a similar OARSI score to that observed in NO-WT mice (Fig. 2a,b).

### GP chondrocyte disorganization evoked by *Evc* deletion does not aggravate AC damage in a DMM-induced OA model

**Figure 3:**
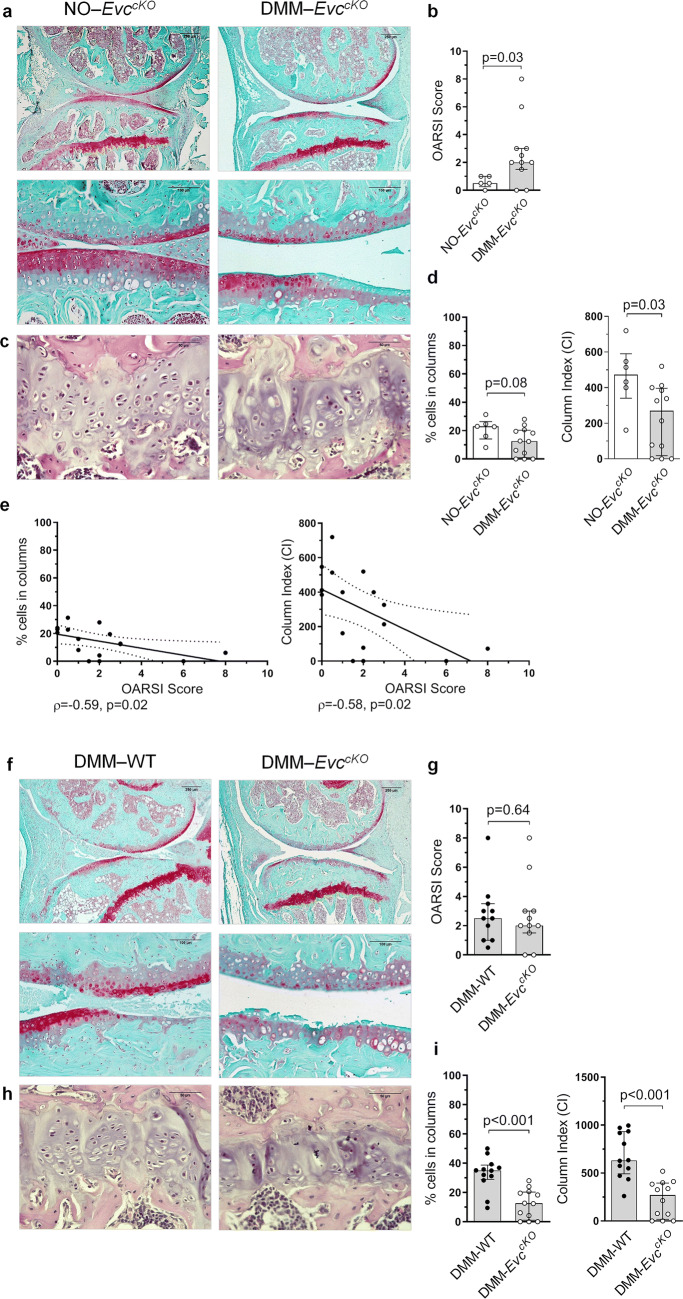
Effect of GP chondrocyte disarrangement on articular cartilage (AC) (**a**) Representative Safranin-O Fast Green stained mice joint sections at × 5 and × 20 magnification in NO-*Evc*^*cKO*^ and DMM-*Evc*^*cKO*^ mice (bars = 250 and 100 µm respectively). (**b**) Histopathological OARSI Score represented as the sum of tibia and femur scores in NO-*Evc*^*cKO*^ and DMM-*Evc*^*cKO*^ mice. (**c**) Representative Haematoxilin-Eosin stained tibia growth plate (GP) sections at × 40 magnification in NO-*Evc*^*cKO*^ and DMM-*Evc*^*cKO*^ mice (bars = 50 µm). (**d**) Percentage of cells in columns and column index (CI) in the GP in NO-*Evc*^*cKO*^ and DMM-*Evc*^*cKO*^ mice. (**e**) Association between the percentage of cells in columns and OARSI score, and between CI and OARSI score in *Evc*^*cKO*^ mice. (**f**) Representative Safranin-O Fast Green stained mice joint sections at × 5 and × 20 magnification in DMM-WT and DMM-*Evc*^*cKO*^ mice (bars = 250 and 100 µm respectively). (**g**) Histopathological OARSI Score represented as the sum of tibia and femur scores in DMM-WT and DMM-*Evc*^*cKO*^ mice. (**h**) Representative Haematoxilin-Eosin stained tibia GP sections at × 40 magnification in DMM-WT and DMM-*Evc*^*cKO*^ mice (bars = 50 µm). (**i**) Percentage of cells in columns and CI in the GP in DMM-WT and DMM-*Evc*^*cKO*^ mice. Data are shown as median ± interquartile range (IQR). NO-WT, n = 10–16; DMM-WT, n = 11–12; NO-*Evc*^*cKO*^, n = 5–6; DMM-*Evc*^*cKO*^, n = 11–12.

In order to establish whether GP alteration could accelerate AC damage evoked by OA, we studied the GP chondrocyte arrangement and the AC histological damage in DMM-*Evc*^*cKO*^ mice. The pattern of disorganization in the GP chondrocytes due to OA was recurrent among these animals. Accordingly, DMM-*Evc*^*cKO*^ mice showed a significantly lower CI than NO-*Evc*^*cKO*^ mice, together with a decreased percentage of chondrocytes organized in columns, although the latter was not statistically significant (Fig. 3c,d).

As expected, DMM surgery induced significant OA-like lesions in the AC of the *Evc*^*cKO*^ mice, as it is shown in the OARSI score assessment (Fig. 3a,b). As described for WT animals, *Evc*^*cKO*^ mice also showed a negative association between the percentage of cells in columns and the OARSI score (ρ = -0.59, p = 0.02), and between the CI and OARSI score (ρ = -0.58, p = 0.02) (Fig. 3e).

DMM-*Evc*^*cKO*^ mice also showed a lower percentage of cells in columns and a lower CI than that observed in DMM-WT mice, exhibiting an absolute loss of chondrocyte organization in the GP (Fig. 3h, i). Additionally, cellularity, total number of columns, and number of cells per column were further decreased in DMM-*Evc*^*cKO*^ mice when compared to DMM-WT animals (Supplementary Fig. S2). However, DMM-WT and DMM-*Evc*^*cKO*^ mice exhibited equally severe lesions in the AC, thus showing a similar OARSI score (Fig. 3f, g). Therefore, the higher disorganization of the GP observed in the DMM-*Evc*^*cKO*^ group did not induce additional deleterious effects on the AC lesions observed in these animals.

At sacrifice, 8 weeks after DMM surgery, all groups showed identical femur and tibia lengths (Supplementary Fig. S3). Furthermore, both cortical and trabecular BMD measured in the femur, tibia and vertebrae, respectively, was similar in the four groups studied (Supplementary Fig. S3), indicating a healthy bone growth and development in genetically modified animals throughout the study. As expected, we observed an increase in BMD in the knee joint of DMM operated animals, which has been described as subchondral bone sclerosis, both in mice^30^ and in OA patients^31^. The increase in knee BMD was similar between DMM-WT and DMM-*Evc*^*cKO*^ mice (Supplementary Fig. S3).

### GP COL-X histological distribution is modified during OA

**Figure 4:**
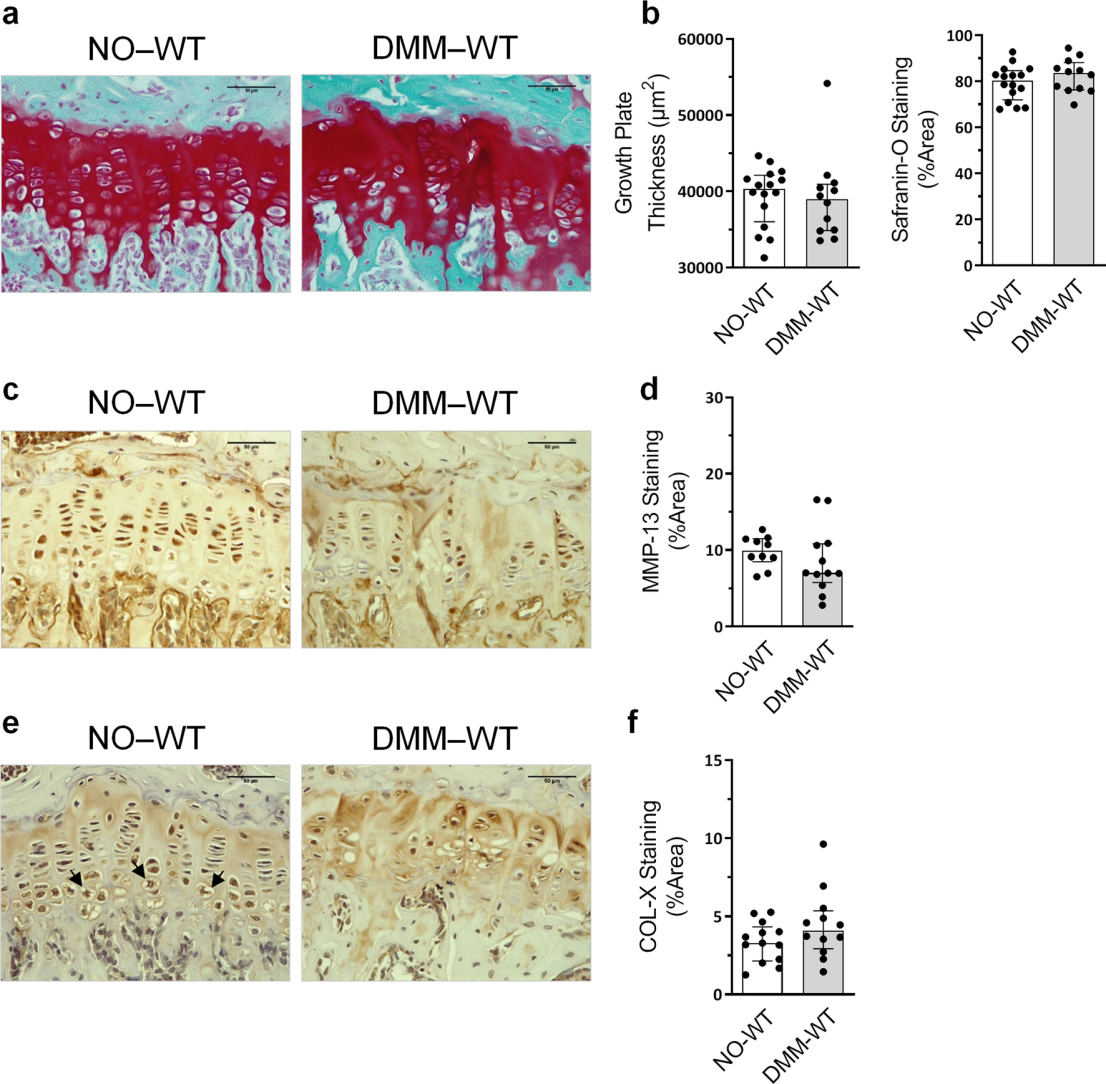
Assessment of the growth plate (GP) pathological state during osteoarthritis (OA). (**a**) Representative Safranin-O Fast Green stained tibia GP sections at × 40 magnification in NO-WT and DMM-WT mice (bars = 50 µm). (**b**) GP thickness and Safranin-O staining quantification. (**c**) Representative MMP-13 immunohistochemistry sections of GP samples (bars = 50 µm). (**d)** MMP-13 staining quantification. (**e**) Representative COL-X immunohistochemistry sections of GP samples (bars = 50 µm). Black arrows indicate GP hypertrophic chondrocytes. (**f**) COL-X staining quantification. Data are shown as median ± interquartile range (IQR). NO-WT, n = 10–16; DMM-WT, n = 12.

The GP thickness was similar in NO-WT and DMM-WT animals, as well as the proteoglycan content in this localization (Fig. 4a, b). Likewise, there was no difference in the amount of MMP-13 and COL-X in the GP between these groups (Fig. 4c–f). However, OA accounted for a certain modification in COL-X localization in the GP, as can be observed in Fig. 4e. In NO-WT, COL-X was mainly located in the ECM of the upper area, where cells exhibited less COL-X staining when compared to the enlarged hypertrophic chondrocytes in the lower area. In DMM-WT mice, an intense ECM staining was observed, and all the cells in the GP seemed to be equally stained (Fig. 4e).

### GP proteoglycan content and COL-X presence are not modified in ***Evc***^***cKO***^ mice

**Figure 5:**
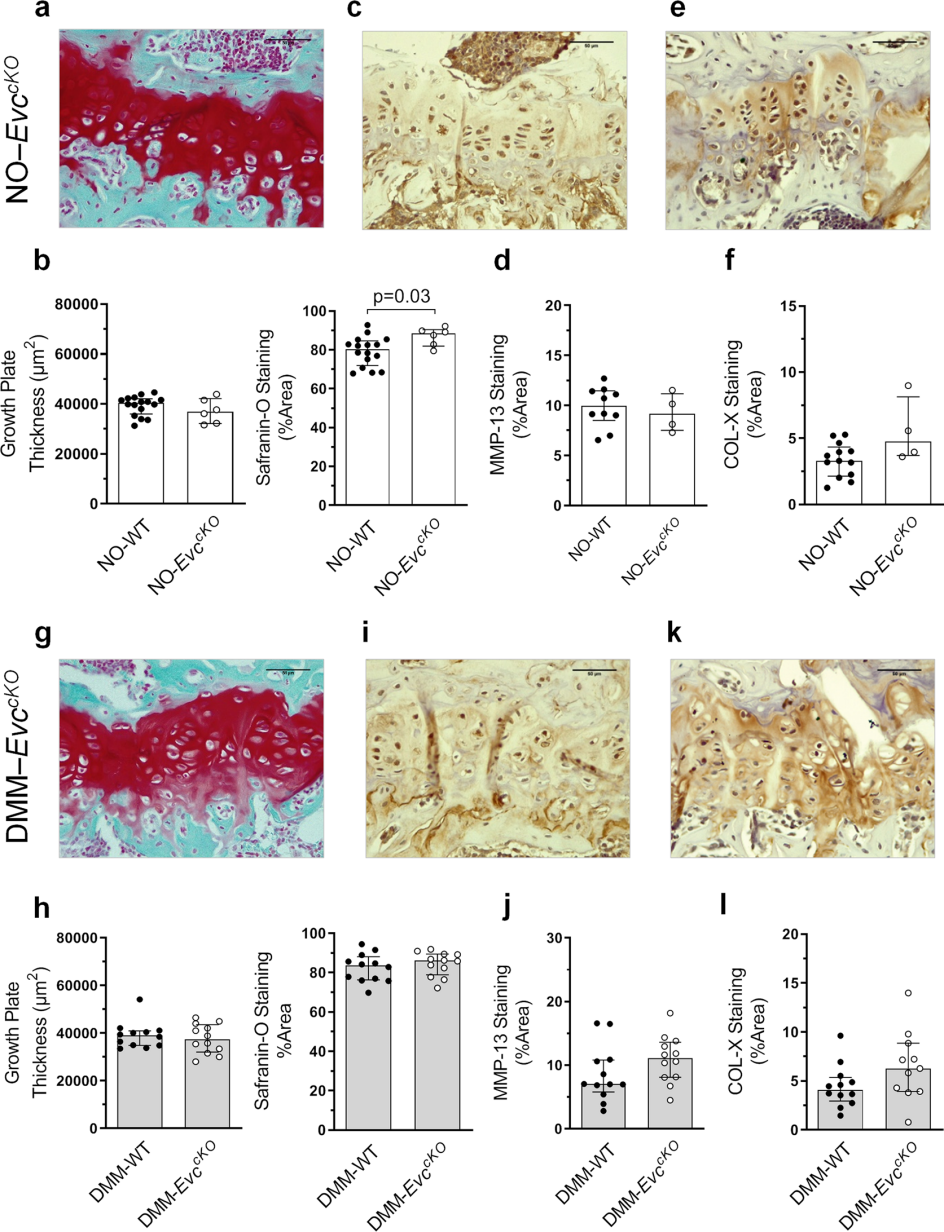
Evaluation of the growth plate (GP) pathological state in *Evc*^*cKO*^ mice. (**a**) Representative Safranin-O Fast Green stained tibia GP sections at × 40 magnification in NO-*Evc*^*cKO*^ mice (bars = 50 µm). (**b**) Comparison of GP thickness and Safranin-O staining quantification in NO-WT and NO-*Evc*^*cKO*^ mice. (**c**) Representative MMP-13 immunohistochemistry of NO-*Evc*^*cKO*^ GP samples (bars = 50 µm). (**d**) Comparison of MMP-13 staining quantification in NO-WT and NO-*Evc*^*cKO*^ mice. (**e**) Representative COL-X immunohistochemistry of NO-*Evc*^*cKO*^ GP samples (bars = 50 µm). (**f**) Comparison of COL-X staining quantification in NO-WT and NO-*Evc*^*cKO*^ mice. (**g**) Representative Safranin-O Fast Green stained tibia GP sections at × 40 magnification in DMM-*Evc*^*cKO*^ mice (bars = 50 µm). (**h**) Comparison of GP thickness and Safranin-O staining quantification in DMM-WT and DMM-*Evc*^*cKO*^ mice. (**i**) Representative MMP-13 immunohistochemistry of DMM-*Evc*^*cKO*^ GP samples (bars = 50 µm). (**j**) Comparison of MMP-13 staining quantification in DMM-WT and DMM-*Evc*^*cKO*^ mice. (**k**) Representative COL-X immunohistochemistry of DMM-*Evc*^*cKO*^ GP samples (bars = 50 µm). (**l**) Comparison of COL-X staining quantification in DMM-WT and DMM-*Evc*^*cKO*^ mice. Data are shown as median ± interquartile range (IQR). NO-WT, n = 10–16; DMM-WT, n = 12; NO-*Evc*^*cKO*^, n = 4–6; DMM-*Evc*^*cKO*^, n = 11–12.

The GP thickness was similar between NO-WT and NO-*Evc*^*cKO*^ mice, although the GP of these animals seemed to contain a higher presence of proteoglycans than that observed in NO-WT (Fig. 5a, b). Both MMP-13 and COL-X staining and localization were comparable in these groups (Fig. 5c–f). DMM surgery did not seem to modify these parameters (Fig. 5g–l).

### GP pathological state may interfere in molecular analyses of AC damage

Our next objective was to assess whether the presence of GP cartilage could disturb the measurement of AC proteins in mouse models that employ the femoral condyle for these studies. As can be observed in Supplementary Figure S4, a significant proportion of the hyaline cartilage comes from the GP when the condyle is isolated for analysis. Therefore, we analysed the presence of AGG and COL-II, two AC proteins usually analysed in OA studies, in mouse condyles with or without AC.

**Figure 6:**
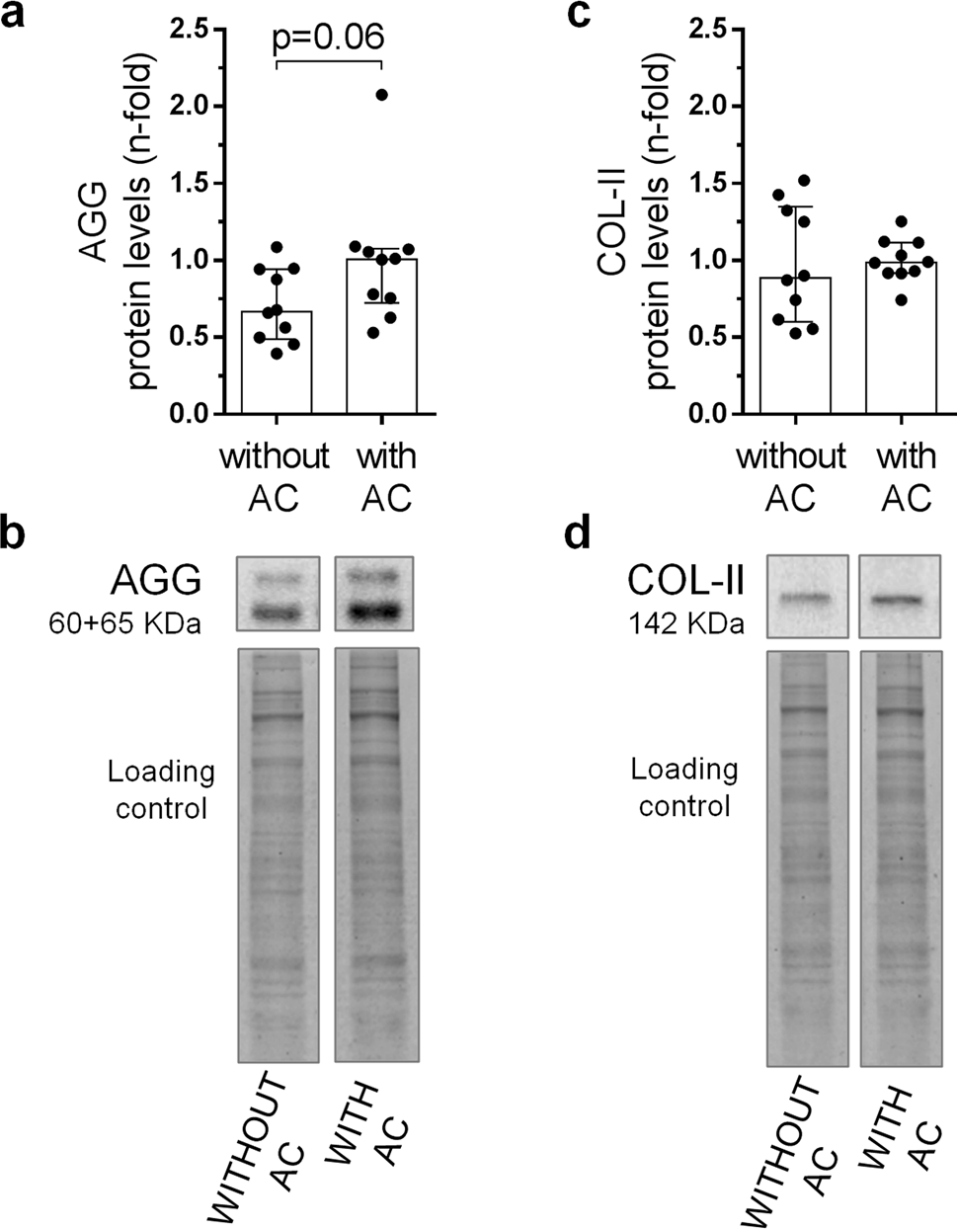
Assessment of growth plate (GP) bias in the molecular analyses of articular cartilage (AC) proteins. (**a**) Protein levels of aggrecan (AGG) in femoral condyles with and without AC of healthy WT mice. (**b**) Western blot analysis of AGG in the mouse femoral condyles. (**c**) Protein levels of type II collagen (COL-II) in femoral condyles with and without AC of healthy WT mice. (**d**) Western blot analysis of COL-II in the mouse femoral condyles. Data are shown as median ± interquartile range (IQR) (n = 10 femora per group).

We observed that femoral condyles without AC showed a tendency of lower AGG protein levels compared to femoral condyles with intact AC, although no statistical significance was observed (Fig. 6a, b). According to these results, a high percentage of total AGG in the mice femoral condyle, around 60–70%, comes from the GP. Furthermore, the amount of COL-II protein in the femoral condyles analysed indicates that around 95% of this protein comes from the GP cartilage when the condyle is studied, since identical COL-II presence was observed in tissues with or without AC (Fig. 6c, d).

## Discussion

A relevant percentage of preclinical studies employing animal models are not reproducible in humans, which results in a worldwide cost overrun in biomedical research^32,33^. For this reason, the search for specific causes that may bias the face value of the results and their interpretation emerges mandatory. Our results show that, besides the damage in the AC, OA induced by knee destabilization in adult mice altered the characteristic columnar structure of the chondrocytes in the GP. Eight weeks after OA induction, columns look shorter and not homogenously organized within the GP, but instead, in irregular clusters. Endochondral ossification is an active process in mice until 10 week-of-age, when bone development concludes and mice show a mature skeleton^9^, even though epiphyses do not ossify in skeletally mature rodents as it happens in humans^18^. In addition, growing mice are resistant to joint damage^9^. In order to abolish any effect due to bone immaturity, we studied GP alterations induced by joint destabilization in 12-week-old mice, once bone growth was completed. Despite being considered a mature GP, our results show that this tissue is profoundly altered by OA in a relatively short period of time. Different causes may be responsible for these modifications, although those related to mechanical alterations seem of great impact. Limb paralysis in neonatal mice that resulted in a reduced mechanical joint loading, also evoked chondrocyte column disorganization at the GP^27^. We contemplate that chondrocyte disorganization and the loss of the column arrangement during DMM-induced OA might be prompted by an alteration of joint biomechanics that results in unbalanced forces. These forces may affect subarticular tissues^34^ and even the GP, and modify cell-to-cell and cell-to-ECM interactions^35^, as well as chondrocyte polarization in the GP.

Both connexins and integrins have been described as mechanotransducers in the cartilage, and modulate cell responses after mechanical stimulation^36,37^, and could therefore be responsible for the alteration in GP structure after joint destabilization. These proteins are especially relevant in chondrocytes, playing an essential role in cellular polarity and structure of the collagen network in the GP, and modulating cell-to cell and cell-to matrix interaction^37–40^. Connexins and integrins can be localized in the chondrocyte primary cilia, where they regulate cell interactions with the surrounding matrix^41–43^.

Once observed the alteration of chondrocyte columns in the GP induced by OA, we wanted to study whether this pathologic disarray could produce AC damage or increase the AC lesions induced by knee destabilization in mice. *Evc*^*-/-*^ embryos and newborn mice had loss of chondrocyte column structure in the GP^26^, together with severe skeletal abnormalities that replicate the human disorder described as EvC syndrome. However, most of them die a few days after birth or cannot feed normally^26^. Here, we have generated a new experimental model for conditional *Evc* deletion in adult mice in order to approach different physio-pathological aspects in OA^26,44^. *Evc*^*cKO*^ mice showed no discernible defects neither at 10 weeks of age, not after TAM-induced complete *Evc* deletion. At 10 weeks of age, *Evc*^*cKO*^ mice showed normal skeletal development, and similar body weight to that observed for their WT littermates, in spite of the loss of *Evc* expression and carrying only one *Evc2* allele (unpublished data), in line with the skeletal properties described for *Evc2*^+*/-*^ heterozygous mice^26,44^. TAM administration in adult mice predictably induced a complete disruption of Hh signaling, according to previous analyses in *Evc*^*-/-*^ embryonic GP^26^. Adult NO-*Evc*^*cKO*^ mice did not show any evident skeletal alteration, their tibia and femur length were normal, and BMD measurements at different bone locations were similar to those observed for WT mice. However, they showed a disorganized GP architecture, with a lesser proportion of chondrocytes in columns to that observed in WT animals. This disorganized GP architecture did not predispose to cartilage damage, since NO-*Evc*^*cKO*^ mice did not exhibit any OA-like lesion in the AC. Furthermore, in DMM-*Evc*^*cKO*^ mice, we observed that knee destabilization induced a higher disorganization in the GP to that observed in DMM-WT or in NO-*Evc*^*cKO*^ mice, although it did not aggravate AC lesions, which were similar to those observed in DMM-WT animals. Therefore, a profound disorganization in the GP did not have any additional detrimental effect on the AC. However, it cannot be discarded that a longer monitoring of these animals, resulting in more severe and chronic joint instability, could presumably display detectable OA-like cartilage lesions, or even the appearance of spontaneous OA.

Since both AC and GP cartilage are damaged during experimental OA, molecular studies using the whole mouse femoral condyle could be misinterpreted due to the presence of a damaged GP. The analyses in these experimental models are not usually performed by the precise isolation of the AC, due to size limitations. Therefore, the whole femoral condyle is commonly employed in gene and protein analyses, which may partially contain GP cartilage. To elucidate this point, we studied the presence and distribution of classic markers of cartilage damage in the GP. While proteoglycan content and MMP-13 presence did not change between healthy and OA WT mice, we observed a different distribution pattern of COL-X in the GP during OA. This result guided our next experiment considering the possibility that molecular analyses employed as usual markers of hyaline cartilage damage may be biased and overestimated by the interference of tissue coming from the GP. We analysed whether the relative presence of AGG and COL-II proteins was different in femoral condyles that contain or not the AC. In both cases, the origin of the protein was mainly the GP, since the lack of the AC did not significantly change the relative presence of these proteins in the femoral condyle analysis. Around 71% AGG and 97% COL-II protein may be originated in the GP. Although the contribution of GP cartilage could be variable between the different samples, our results showed that molecular analysis of the cartilage in mice samples containing the GP should be interpreted with caution.

The limitations of this study include the impossibility of assessing the GP bias on AC during OA, due to the unfeasible duplication of the experimental model to obtain the femoral condyles for this purpose.

In conclusion, surgical knee destabilization in mice is responsible for an altered GP structure, accounting for chondrocyte disorganization and loss of column arrangement, although this alteration in the GP does not predispose nor aggravate the development of more severe damage in AC of the affected joint. AC damage and GP disarrangement could be similarly triggered by joint instability, but both seem independent phenomena in terms of their progression and underlying mechanisms. Since adult mice exhibit such a great affectation of the GP structure, molecular measurements might be overestimated by protein content dragged from the GP when employing the whole condyle in order to study AC during OA. Additional research would be necessary to determine this interference and its significance in mouse OA model, in order to prevent bias in experimental models that may subsequently cause a lack of reproducibility in the human disease.

## Methods

### Animal model of OA

OA was induced in 12-week old female WT mice by DMM as previously described^8^ (DMM-WT, n = 12 joints), while non-operated WT mice were simultaneously studied (NO-WT, n = 16 joints) (Supplementary Fig. S1).

Generation of TAM-induced *Evc* knockouts was achieved by crossing mice carrying an *Evc* floxed allele (*Evc*^*flox/*+^*)*^44^ with UBC-*CreERT2* (*Tg.hUBC-CreERT2*) transgenics^45^. Male offspring of these crosses that were heterozygous for both CreERT2 and the *Evc* floxed allele were subsequently mated to females heterozygous for an *Evc* null allele (*Evc*^+*/-*^)^26^ to obtain *Evc*^*flox/-*^*;UBC-CreERT2* mice. The generation of the *Evc* floxed allele and the *Evc* null allele have previously been described by *Caparros-Martin *et al. and *Ruiz-Perez *et al. respectively^26,44^. Of note, the *Evc* floxed allele is also null for *Evc2* (Supplementary Fig. S1)^26^. Mice were maintained in a C57BL/6;129 mixed background. At 10 weeks of age^9^, *Evc*^*flox/-*^;*UBC-CreERT2*^+*/-*^ female mice received 0.075 mg/g/day TAM administered in five intraperitoneal injections. These mice will be referred to as *Evc*^*cKO*^ and were used as a model of chondrocyte disorganization in the GP. OA was induced in 12-weeks old *Evc*^*cKO*^ mice by DMM (DMM-*Evc*^*cKO*^, n = 12 joints), while non-operated *Evc*^*cKO*^ mice were simultaneously followed (NO-*Evc*^*cKO*^, n = 6 joints).

*Evc*^*cKO*^ littermates that were genotyped with *Evc* and *Evc2* WT alleles were used as WT animals in the OA model.

Eight weeks following DMM surgery, all mice were anesthetized and bone mineral density (BMD) was assessed by dual-energy X-ray absorptiometry using PIXImus (GE Lunar Corp., USA). Afterwards, mice were euthanized and knee joints fixed in 4% formaldehyde (Sigma-Aldrich, USA), decalcified and embedded in paraffin for histopathological analyses (Supplementary Fig. S1). Animal handling, experimental protocol PROEX 119/16 and all methods complied with Spanish Regulations and the Guidelines for the Care and Use of Laboratory Animals drawn up by the National Institutes of Health (Bethesda, MD, USA). Experimental protocols were approved by the Institutional Ethics and Welfare Committees of the IIS-Fundación Jiménez Díaz and Alberto Sols Biomedical Research Institute.

### Analysis of the interference of GP cartilage proteins in the study of AC proteins

Fifteen 3-month-old female C57BL/6 WT mice were euthanized and the femurs were dissected. The AC from left femoral condyles was scraped with a surgical scalpel until completely removed, while right femora were kept intact. Femoral condyle samples from 10 mice were immediately frozen in liquid N_2_ for protein analysis, while the remaining 5 samples were fixed in 4% formaldehyde (Sigma-Aldrich), decalcified and embedded in paraffin for histological analyses.

### GP chondrocyte column organization assessment

The analysis of the GP chondrocyte column organization was performed in the knee sections obtained eight weeks after DMM surgery from operated and non-operated animals. 4 µm sagittal paraffin-embedded knee sections were stained with Haematoxylin–Eosin. Joint sections were photographed with a Leica DM3000 LED digital micro-imaging instrument (Leica, Microsystems, Inc. Buffalo Grove, IL, USA) at the central region of the tibia at × 40 magnification, and chondrocyte histomorphometry was evaluated as described elsewhere^27,46^. Briefly, a chondrocyte column was defined as a chondron containing a minimum of three chondrocytes, located no more than 20 pixels apart one from each other, and with angles between them ranging from -155° to -179° or 155° to 180°. These parameters were computed as previously described by *Killion et al*^27^. Once the columns were established in each sample, the number of cells was quantified using the Image J Software (NIH, USA) cell counter plug-in. Then, the percentage of cells in columns was obtained by calculating the ratio ‘cells in columns’/ ‘total cells in the GP’. Column Index (CI) was calculated multiplying the percentage of cells in columns by their respective length^27^.

### Histopathological assessment of the cartilage

AC was evaluated in 4 µm sagittal knee joint sections that were stained with Safranin-O Fast Green. The histological damage was assessed by two blinded observers following the OARSI recommendations for mice samples^47^, which were adapted for sagittal slices. The semi-quantitative 0 to 6 points scoring system was applied to one area in the femur and one area in the tibia, and the sum of these two scores was represented as the total score.

Proteoglycan content in the GP cartilage was also evaluated in Safranin-O Fast Green stained joint samples. Sections were photographed with a Leica DM3000 LED digital micro-imaging instrument (Leica, Microsystems, Inc. Buffalo Grove, IL, USA) at the central region of the tibia at × 40 magnification. Specific Safranin-O staining in the femur and tibia GP, as well as GP thickness, were measured employing Image J software, and corrected by sample area.

### Immunohistochemical analysis

4 µm knee joint sections were deparaffinized and rehydrated. Antigen retrieval was performed by incubation with 20 µg/mL proteinase K (Promega, USA). Tissue sections were processed as previously described^48–50^, and then incubated with the corresponding primary antibody: anti-mouse type X collagen (COL-X) (1/2000; ab58632, Abcam, UK) or anti-metalloproteinase (MMP)-13 (1/200; ab39012; Abcam). A secondary biotinylated anti-rabbit IgG was used for detection of positive signal through a horseradish peroxidase linked to an avidin/biotin complex (ABC) (Vector Laboratories, USA) using 3,3 diaminobenzidine tetra-hydrochloride as chromogen (Dako, Denmark). Joint sections were counterstained with Haematoxylin, dehydrated and mounted in DPX (Merck Millipore, USA). Sections were photographed with a Leica DM3000 LED digital micro-imaging instrument (Leica, Microsystems, Inc. Buffalo Grove, IL, USA) at the central region of the tibia at × 40 magnification, and specific staining in the GP was quantified using Image J software and corrected by sample area.

### Dual-energy X-ray absorptiometry

BMD (g/cm^2^) was assessed eight weeks after surgery in anesthetized mice, by dual-energy X-ray absorptiometry (DXA) using PIXImus (GE Lunar Corp., USA), as previously described^51^. BMD was assessed in different regions of interest (ROIs). For subchondral BMD, we employed a 11 × 11 pixel ROI focused on the knee joint; cortical BMD was measured in the femur and in the tibia employing a 17 × 13 pixel ROI; and trabecular bone BMD was evaluated on L2 and L3 lumbar vertebrae employing a 35 × 23 pixel ROI. Images were scaled according to the PIXImus phantom size, and femur and tibia lengths were measured employing the Image J software.

### Western blot

Briefly, 20 μg of total protein from tissues were extracted as described elsewhere^50^, separated by electrophoresis in SDS–polyacrylamide gels and transferred to a nitrocellulose membrane in a semi-dry Trans-Blot device (Bio-Rad, Madrid, Spain). The following primary antibodies were applied: anti-mouse aggrecan (AGG) antibody (1/100; ab3778; Abcam) and anti-mouse type II collagen (COL-II) antibody (1/1,000; ab34712; Abcam) in 3% BSA, overnight at 4 °C. The binding signal was detected by chemiluminescence using Horseradish Peroxidase-linked secondary antibodies. EZBlue gel staining reagent (Sigma-Aldrich) was used for tissue protein loading control. Densitometric measurements were normalized relative to total protein presence in femoral condyles with AC using Quantity One software (Bio-Rad) and expressed as arbitrary densitometric units (A.U.)^49,50,52^.

### Statistical analysis

Each limb was considered as an independent sample. Based on previously published data^27,48^, the sample size was determined on the expected difference to be detected regarding the percentage of cells in columns. By accepting a significance level (alpha) of 5%, and a statistical power of 80%, a pairwise t-test between conditions requires 6 limbs per group to demonstrate an approximate 35% change (70 vs 25) assuming a standard deviation of 5, calculated using the G-power 3.1 software (G-power, Germany)^53^. Owing to the generation limitations for *Evc*^*cKO*^ mice, the required number of samples obtained by statistical prediction was assigned to the NO-*Evc*^*cKO*^ group (n = 6 limbs). The n was tripled for the DMM-*Evc*^*cKO*^ group (n = 18 limbs), to ensure the required number in case of unexpected deaths. During surgery two mice died due to a defective assimilation of anaesthesia, and another one shortly after surgery, remaining n = 12 limbs. The n assigned to NO-WT and DMM-WT groups was 16 limbs, due to lack of survival limitations. However, two mice died a few days after surgery, remaining 12 limbs in the DMM-WT group.

No adjustment for multiple comparisons between groups was made a priori, but to preserve type one error, individual tests were only undertaken following a significant omnibus ANOVA test. Non-parametric tests were used due to a lack of normality based on a Kolmogorov–Smirnov test. Kruskal–Wallis test was used for comparisons between multiple groups prior to Mann–Whitney U-test for comparisons between two groups, adopting a Bonferroni correction. A P-value of less than 0.05 was considered statistically significant. Associations were expressed using Spearman's rank correlation coefficient (rho, ρ). Statistical analyses of data were performed using GraphPad Prism 6.01 for Windows (GraphPad Software, USA). Data were expressed as median with interquartile range (IQR).

## Supplementary information

Supplementary file1 (PDF 891 kb)

## Data Availability

All data generated or analysed during this study are included in this published article (and its Supplementary Information files).

## References

[1] Loeser RF, Goldring SR, Scanzello CR, Goldring MB (2012). Osteoarthritis: A disease of the joint as an organ. Arthritis Rheum..

[2] Martel-Pelletier J (2016). Osteoarthritis. Nat. Rev. Dis. Prim..

[3] Guilak F (2011). Biomechanical factors in osteoarthritis. Best Pract. Res. Clin. Rheumatol..

[4] Johnson VL, Hunter DJ (2014). The epidemiology of osteoarthritis. Best Pract. Res. Clin. Rheumatol..

[5] Herrero-Beaumont G (2019). Targeting chronic innate inflammatory pathways, the main road to prevention of osteoarthritis progression. Biochem. Pharmacol..

[6] O’Neill TW, McCabe PS, McBeth J (2018). Update on the epidemiology, risk factors and disease outcomes of osteoarthritis. Best Pract. Res. Clin. Rheumatol..

[7] Goldring MB, Goldring SR (2007). Osteoarthritis. J. Cell. Physiol..

[8] Glasson SS, Blanchet TJ, Morris EA (2007). The surgical destabilization of the medial meniscus (DMM) model of osteoarthritis in the 129/SvEv mouse. Osteoarthr. Cartil..

[9] Lorenz J, Grässel S (2014). Experimental osteoarthritis models in mice. Methods Mol. Biol..

[10] O’Brien M, Philpott H, McDougall J (2017). Understanding osteoarthritis pain through animal models. Clin. Exp. Rheumatol..

[11] Cornelis F (2019). Increased susceptibility to develop spontaneous and post-traumatic osteoarthritis in Dot1l-deficient mice. Osteoarthr. Cartil..

[12] Wang X (2019). MicroRNA-21-5p as a novel therapeutic target for osteoarthritis. Rheumatol..

[13] Kuyinu EL, Narayanan G, Nair LS, Laurencin CT (2016). Animal models of osteoarthritis: Classification, update, and measurement of outcomes. J. Orthop. Surg. Res..

[14] Chagin AS (2004). Estrogen receptor-β inhibits skeletal growth and has the capacity to mediate growth plate fusion in female mice. J. Bone Miner. Res..

[15] Börjesson AE (2010). The role of estrogen receptor α in growth plate cartilage for longitudinal bone growth. J. Bone Miner. Res..

[16] Schlegel W (2010). IGF expression patterns and regulation in growth plate chondrocytes. Mol. Cell. Endocrinol..

[17] Emons J, Chagin AS, Sävendahl L, Karperien M, Wit JM (2011). Mechanisms of growth plate maturation and epiphyseal fusion. Horm. Res. Paediatr..

[18] Jilka RL (2013). The relevance of mouse models for investigating age-related bone loss in humans. J. Gerontol. Ser. A Biol. Sci. Med. Sci..

[19] The Economist Group Limited. The world’s favourite lab animal has been found wanting, but there are new twists in the mouse’s tale. *Econ.* Christmas Special Edition (2016).

[20] Bovée JVMG, Hogendoorn PCW, Wunder JS, Alman BA (2010). Cartilage tumours and bone development: Molecular pathology and possible therapeutic targets. Nat. Rev. Cancer.

[21] Li J, Dong S (2016). The signaling pathways involved in chondrocyte differentiation and hypertrophic differentiation. Stem Cells Int..

[22] Ohba S (2016). Hedgehog signaling in endochondral ossification. J. Dev. Biol..

[23] De Andrea CE (2010). Primary cilia organization reflects polarity in the growth plate and implies loss of polarity and mosaicism in osteochondroma. Lab. Investig..

[24] Zhang W (2018). Expanding the genetic architecture and phenotypic spectrum in the skeletal ciliopathies. Hum. Mutat..

[25] Ibarra-Ramírez M (2017). Phenotypic variation in patients with homozygous c.1678G>T mutation in EVC gene report of two mexican families with Ellis-van Creveld syndrome. Am. J. Case Rep..

[26] Ruiz-Perez VL (2007). Evc is a positive mediator of Ihh-regulated bone growth that localises at the base of chondrocyte cilia. Development.

[27] Killion CH, Mitchell EH, Duke CG, Serra R (2017). Mechanical loading regulates organization of the actin cytoskeleton and column formation in postnatal growth plate. Mol. Biol. Cell.

[28] Park H, Kong SY, Kim HW, Yang IH (2012). Altered cellular kinetics in growth plate according to alterations in weight bearing. Yonsei Med. J..

[29] Esapa CT (2012). A mouse model for spondyloepiphyseal dysplasia congenita with secondary osteoarthritis due to a Col2a1 mutation. J. Bone Miner. Res..

[30] Lories RJ, Luyten FP (2011). The bone-cartilage unit in osteoarthritis. Nat. Rev. Rheumatol..

[31] Bousson V, Lowitz T, Laouisset L, Engelke K, Laredo JD (2012). CT imaging for the investigation of subchondral bone in knee osteoarthritis. Osteoporos. Int..

[32] Richter SH (2017). Systematic heterogenization for better reproducibility in animal experimentation. Lab Anim..

[33] Laman JD, Kooistra SM, Clausen BE (2017). Reproducibility issues: avoiding pitfalls in animal inflammation models. Methods Mol. Biol..

[34] Felson DT (2013). Osteoarthritis as a disease of mechanics. Osteoarthritis and Cartilage.

[35] Martínez-Moreno D, Jiménez G, Gálvez-Martín P, Rus G, Marchal J (2019). Cartilage biomechanics: A key factor for osteoarthritis regenerative medicine. Biochim. Biophys. Acta Mol. Basis Dis..

[36] Ramage L, Nuki G, Salter DM (2009). Signalling cascades in mechanotransduction: cell-matrix interactions and mechanical loading. Scand. J. Med. Sci. Sport..

[37] Donahue HJ, Qu RW, Genetos DC (2017). Joint diseases : from connexins to gap junctions. Nat. Rev. Rheumatol..

[38] Prein C, Beier F (2019). ECM Signaling in Cartilage Development and Endochondral Ossification. Current Topics in Developmental Biology.

[39] DeLise AM, Fischer L, Tuan RS (2000). Cellular interactions and signaling in cartilage development. Osteoarthr. Cartil..

[40] Varela-Eirín M (2018). Targeting of chondrocyte plasticity via connexin43 modulation attenuates cellular senescence and fosters a pro-regenerative environment in osteoarthritis. Cell Death Dis..

[41] Mcglashan SR, Jensen CG, Poole CA (2006). Localization of extracellular matrix receptors on the chondrocyte primary cilium. J. Histochem. Cytochem..

[42] McGlashan SR, Haycraft CJ, Jensen CG, Yoder BK, Poole CA (2007). Articular cartilage and growth plate defects are associated with chondrocyte cytoskeletal abnormalities in Tg737orpk mice lacking the primary cilia protein polaris. Matrix Biol..

[43] Knight MM, Mcglashan SR, Garcia M, Jensen CG, Poole CA (2009). Articular chondrocytes express connexin 43 hemichannels and P2 receptors—A putative mechanoreceptor complex involving the primary cilium ?. J. Anat..

[44] Caparros-Martin JA (2013). The ciliary Evc / Evc2 complex interacts with Smo and controls Hedgehog pathway activity in chondrocytes by regulating Sufu/Gli3 dissociation and Gli3 trafficking in primary cilia. Hum. Mol. Genet..

[45] Ruzankina Y (2007). Deletion of the developmentally essential gene ATR in adult mice leads to age-related phenotypes and stem cell loss. Cell Stem Cell.

[46] Randall RM, Shao YY, Wang L, Ballock RT (2012). Activation of Wnt Planar cell polarity (PCP) signaling promotes growth plate column formation in vitro. J. Orthop. Res..

[47] Glasson SS, Chambers MG, Van Den Berg WB, Little CB (2010). The OARSI histopathology initiative - recommendations for histological assessments of osteoarthritis in the mouse. Osteoarthr. Cartil..

[48] Gratal P (2019). Chondrocyte enlargement is a marker of osteoarthritis severity. Osteoarthr. Cartil..

[49] Andrés-Bergós J (2012). The increase in O-linked N-acetylglucosamine protein modification stimulates chondrogenic differentiation both in vitro and in vivo. J. Biol. Chem..

[50] Pérez-baos S (2019). Inhibition of pSTAT1 by tofacitinib accounts for the early improvement of experimental chronic synovitis. J Inflamm (Lond).

[51] Estañ MC (2019). Recessive mutations in muscle-specific isoforms of FXR1 cause congenital multi-minicore myopathy. Nat. Commun..

[52] Larrañaga-Vera A (2017). Increased synovial lipodystrophy induced by high fat diet aggravates synovitis in experimental osteoarthritis. Arthritis Res. Ther..

[53] Faul F, Erdfelder E, Lang A-G, Buchner A (2007). G * Power 3: A flexible statistical power analysis program for the social, behavioral, and biomedical sciences. Behav. Res. Methods.

