# Supplementary material for: Disorganization of chondrocyte columns in the growth plate does not aggravate experimental osteoarthritis in mice
Source: Sci Rep. 2020 Jul 1;10:10745. doi: 10.1038/s41598-020-67518-0 (PMC7329885; doi:10.1038/s41598-020-67518-0)
Supplement: Supplementary file 1 — Supplementary file1 (PDF 891 kb) [file 41598_2020_67518_MOESM1_ESM.pdf]

Disorganization of Chondrocyte Columns in the Growth Plate does not Aggravate Experimental Osteoarthritis in Mice

Ana Lamuedra[1], Paula Gratal[1], Lucía Calatrava[2,3], Víctor Luis Ruiz-Perez[2,3,4], Raquel Largo*[1], Gabriel Herrero-Beaumont[1]

[1] Bone and Joint Research Unit. Rheumatology Department. IIS-Fundación Jiménez Díaz UAM, 28040 Madrid, Spain

[2] Instituto de Investigaciones Biomédicas 'Alberto Sols', CSIC-UAM, 28029 Madrid, Spain

[3] CIBER de Enfermedades Raras (CIBERER), ISCIII, Spain

[4] Instituto de Genética Médica y Molecular (INGEMM), Hospital Universitario La Paz-IdiPaz-UAM, 28046 Madrid, Spain

***Correspondence**
Raquel Largo, PhD
Bone and Joint Research Unit
IIS-Fundacion Jimenez Diaz
Reyes Católicos, 2
28040 Madrid Spain
Telephone +34 915 504 978
rlargo@fjd.es

Ana Lamuedra: ana.lamuedra@quironsalud.es
Paula Gratal: paula.gratal@fjd.es
Lucía Calatrava: luciacalatrava@gmail.com
Víctor Luis Ruiz-Perez: vlruiz@iib.uam.es
Raquel Largo: rlargo@fjd.es
Gabriel Herrero-Beaumont: gherrero@fjd.es

Supplementary Figure S1

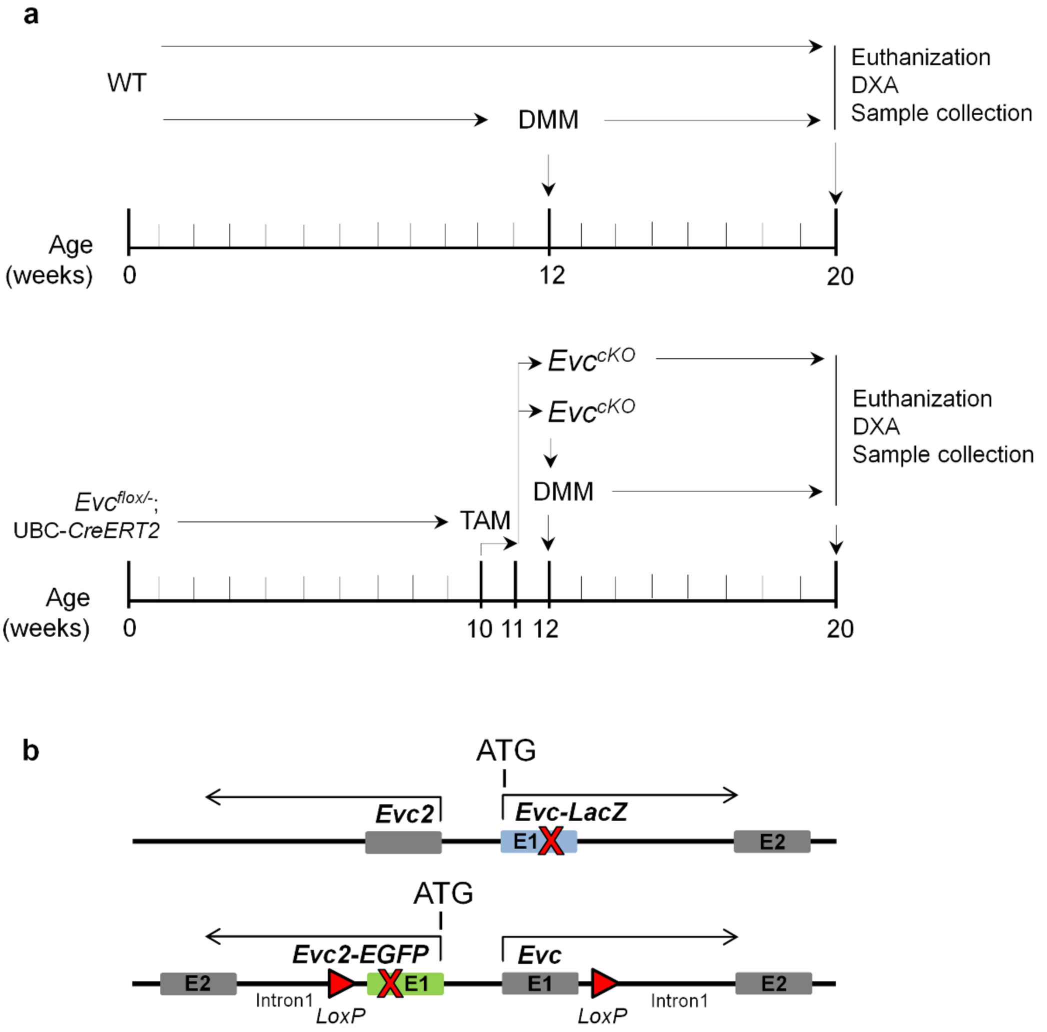

Supplementary Figure S1. Experimental model of knee osteoarthritis (OA) by destabilization of the medial meniscus (DMM) in mice. (a) Chronogram of the experimental OA model. (b) Representation of allele composition of *Evc*$^{flox/-}$*; UBC-CreERT2* mice. In comparison to wild-type (WT) mice, *Evc*$^{flox/-}$ mutants have one copy of *Evc* inactive and the other one flanked by *LoxP* sites. The *Evc* null allele was generated by replacing exon1 of this gene by the *LacZ* reporter. Similarly, the first exon of *Evc2* was replaced by *EGFP* in the *Evc* floxed allele, while two *LoxP* sites (red triangles) were inserted in the intron 1 of each gene. Thus *Evc*$^{flox/-}$ mice only have one active copy of *Evc2*.

Supplementary Figure S2

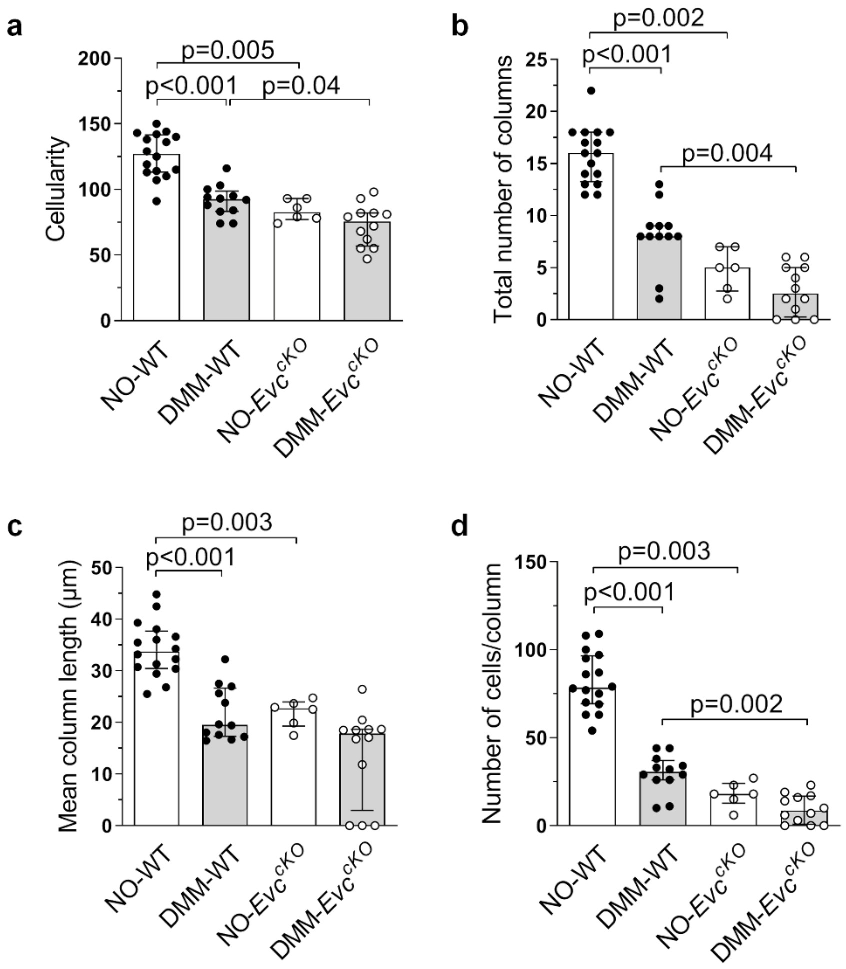

Supplementary Figure S2. Additional column measurements in the growth plate (GP). (a) Total number of cells (cellularity), (b) total number of columns, (c) mean column length and (d) number of cells per column in the growth plate (GP). Data are expressed as median ± interquartile range (IQR). NO-WT n=16; DMM-WT n=12; NO-*Evc*^{cKO} n=6; DMM-*Evc*^{cKO} n=12.

Supplementary Figure S3

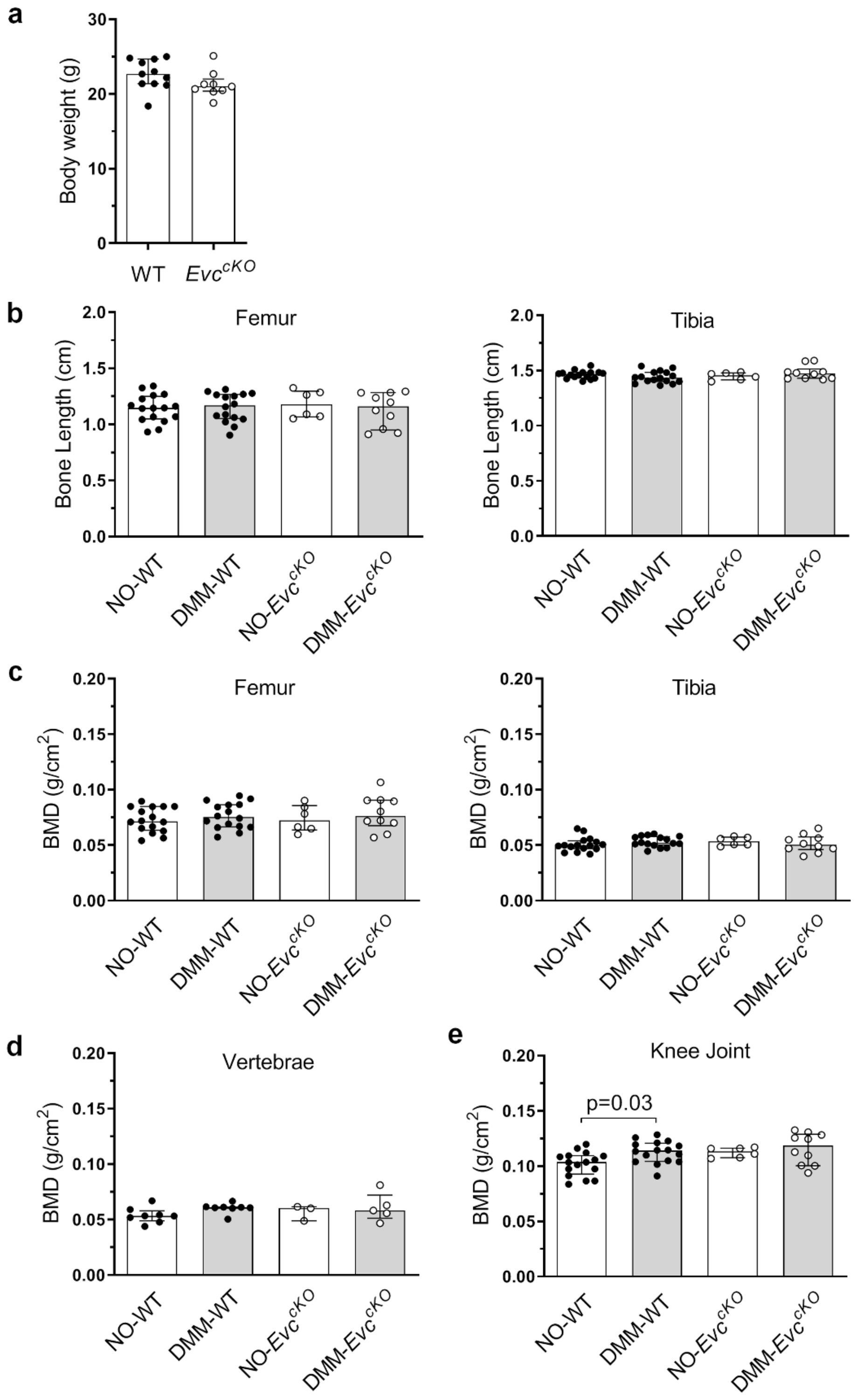

Supplementary Figure S3. Bone growth and quality assessment. (a) Mice Body weight at 12 weeks of age, just before DMM surgery in WT and *Evc*^{cKO} mice (WT n=11; *Evc*^{cKO} n=9). (b) Femur and tibia length at the end of the study, 8 weeks after DMM surgery. (c)

Cortical BMD in femur and tibia at the end of the study. (d) Trabecular BMD in L2 and L3 vertebrae at the end of the study. (e) Bone mineral density (BMD) in knee joint at the end of the study. Data are shown as median ± interquartile range (IQR). NO-WT n=8; DMM-WT n=8; NO-*Evc*^{cKO} n=3; DMM-*Evc*^{cKO} n=5 mice for vertebrae BMD measurements; NO-WT n=16; DMM-WT n=16; NO-*Evc*^{cKO} n=6; DMM-*Evc*^{cKO} n=10 limbs for knee joint and femur and tibia measurements.

Supplementary Figure S4

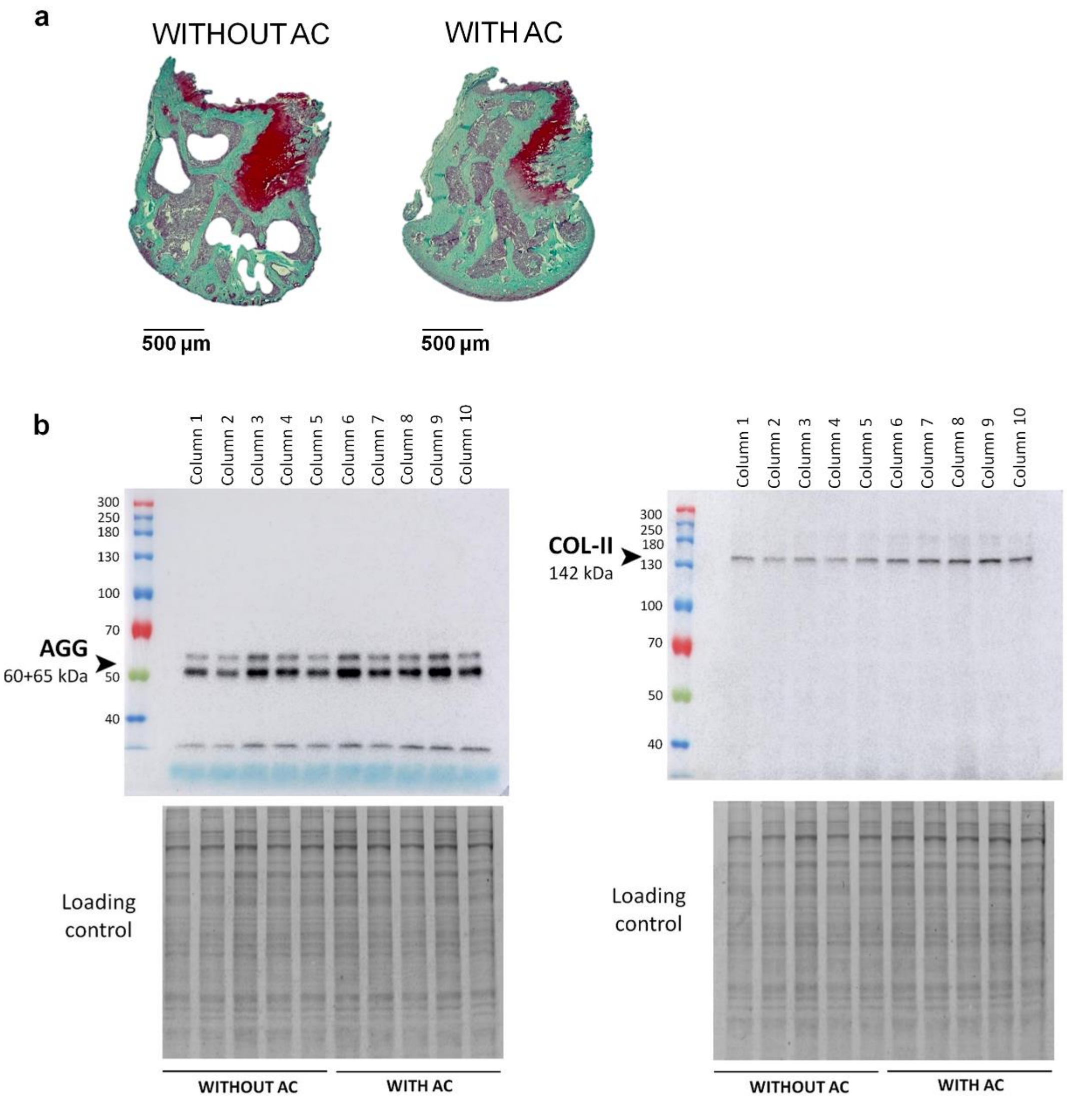

Supplementary Figure S4. Additional data referred to Figure 6. (a) Representative Safranin-O Fast Green stained femoral condyles sections with and without articular cartilage (AC) at x 2.5 magnification (bars=500 µm). (b) Full length western blot of aggrecan (AGG) and type II collagen (COL-II) in femoral condyles with and without AC of healthy WT mice, with reference to Figure 6. EZ Blue was used as loading control. Columns 2 and 7 of the AGG western blot and loading control gel were selected as representative blots for Figure 6b. Columns 5 and 7 of the COL-II western blot and loading control gel were selected as representative blots for Figure 6d.
